# Awareness and Knowledge Among Dental and Medical Undergraduate Students Regarding Human Papilloma Virus and Its Available Preventive Measures

**DOI:** 10.5334/aogh.2826

**Published:** 2020-11-25

**Authors:** Mayithiri Balaji, Arun Panwar, M. Adarsh Kudva, N. Vasudev Ballal, Vaishali Keluskar

**Affiliations:** 1 Manipal College of Dental Sciences, Manipal, IN

## Abstract

**Background:**

India is a major contributor to the global burden of human papilloma virus (HPV) infection and associated diseases like cervical and oropharyngeal cancers. Hence, it is essential to recognize the existing knowledge pool of current healthcare students about HPV and its preventive measures to translate this into benefits for the society in the future.

**Objective:**

To determine the awareness and knowledge among dental and medical undergraduate students regarding HPV and its diagnosis and prevention.

**Methods:**

This cross-sectional study enrolled in 577 dental and undergraduate medical students from a tertiary-care teaching hospital. A questionnaire containing 20 closed-ended multiple-choice questions was used to assess their knowledge regarding HPV and its transmission, cervical cancer and its screening, as well as HPV vaccines and their attitude towards them. Descriptive statistics, Mann Whitney U test, and Chi square test were employed for statistical analysis. p ≤ 0.05 was considered statistically significant.

**Results:**

The study consisted of 52.68% dental and 47.31% medical students, with a mean age of 20.95 ± 1.82 years, M:F ratio of 0.7:1, and a mean overall score of 10.75 ± 5.18 (average). The mean scores for knowledge about HPV, its vaccination, and its diagnosis were 7.98 ± 3.26 (good), 1.61 ± 0.95 (average), and 1.15 ± 1.16 (average), respectively. These scores showed no significant difference between the courses as well as the genders (p > 0.05).

**Conclusion:**

Overall, the dental and medical undergraduate students presented an average level of knowledge and awareness regarding HPV and its prevention. This reflects a greater need for educating healthcare professionals in order to have a ripple effect on society at large.

## Introduction

Human papilloma virus (HPV) is a high-risk virus known to cause many diseases like warts, cervical cancer (CC), vulvar cancer, and oropharyngeal cancer, spread by physical or sexual contact [1,2]. Globally, CC is the fourth most common cancer among women and is responsible for 7.5% of all female cancer deaths [1]. In India, CC accounts for up to 43.8% of all cancers among women, more than 80% of which are associated with HPV types 16 and 18 [3,4,5]. These strains are also responsible for nearly 92% of anal cancers, 89% of oropharyngeal cancers and 63% of penile cancers in men [6]. Hence, HPV vaccines are approved and recommended before sexual debut for both the genders [1,6,7]. The United States Food and Drug Administration (USFDA)-approved and commercially available HPV vaccines in India include Gardasil (quadrivalent) and Cervarix (bivalent) [8].

Despite safe formulation and effective immunization regimens through various studies, HPV vaccination does not find a place in the National Immunization Program of India, mainly due to financial concerns [9,10]. In such a scenario, widespread public screening becomes essential for control of HPV infection [1,9]. The current World Health Organization (WHO)-recommended screening protocols include HPV testing for high-risk HPV types, visual inspection with acetic acid (VIA), Papanicolaou (pap) smear test, and liquid-based cytology (LBC) [1].

Healthcare professionals (HCP) play a critical role in spreading awareness about these screening and vaccinations and helping patients and society overcome the socio-cultural, religious, and ethical stigmas associated with them [7]. However, lack of updated knowledge among HCP poses a barrier to creating awareness about and accessing these anti-HPV services [7]. Since HPV causes both oropharyngeal and genital lesions, it requires efforts by both dental and medical doctors for its elimination [1,2]. It is best to incorporate this information and acceptance early in the shaping of their medical knowledge since current students would be future doctors [[Bibr B11][Bibr B12][Bibr B13]. However, most studies evaluating awareness and attitude towards HPV have focussed on medical students, but not on dental students [11,12,13].

Hence, the present research was initiated as the first to determine the awareness and knowledge among dental and medical undergraduate students regarding HPV and its diagnosis and prevention.

## Materials and Methods

This cross-sectional questionnaire-based study was conducted at tertiary care teaching hospital in Belagavi, Karnataka, India, from November 2018 to February 2019, after obtaining ethical clearance from the Institutional Review Board.

The study enrolled 577 dental and undergraduate medical students from this teaching hospital, after randomly approaching them and obtaining written informed consent from them, assuring the confidentiality of their responses.

A questionnaire containing 20 closed-ended multiple-choice questions was distributed among the participants, assessing their knowledge of HPV and its mode of transmission, cervical cancer, and its screening, as well as HPV vaccines and their attitude towards them. The participants were instructed to tick the most appropriate choice according to them and then return the questionnaires with their marked choices for calculation.

### Statistical analysis

Data was compiled & descriptive statistical analysis performed using statistical software R 3.6.3 and Microsoft Excel. Each item of the questionnaire was analyzed with descriptive statistics, frequencies, and percentages. Overall, the items with their relative percentages of awareness were tabulated. Continuous variables were represented by mean ± standard deviation (SD) form. Categorical variables were represented by frequency tables. Mann Whitney U test was done to compare two groups for continuous variables. Chi square test was performed to evaluate the association between two categorical variables. p ≤ 0.05 was considered statistically significant.

## Results

The study included 577 students with a mean age of 20.95 ± 1.82 years and an M:F ratio of 0.7:1. Table 1 presents the descriptive statistics for categorical and continuous variables. The study consisted of 52.68% dental and 47.31% medical students, with a mean overall score of 10.75 ± 5.18 (average). The mean scores for knowledge about HPV, its vaccination, and its diagnosis were 7.98 ± 3.26 (good), 1.61 ± 0.95 (average), and 1.15 ± 1.16 (average), respectively.

**Table 1:** Descriptive statistics for categorical and continuous variables.

Variable		Frequency (%) [n = 577]	Mean ± SD	Median (IQR)

Gender	Female	336 (58.23)	–	–
	Male	241 (41.76)		
Course	Dental (BDS)	304 (52.68)	–	–
	Medical (MBBS)	273 (47.31)		
Overall total score	Average (≤10)	294 (50.95)	10.75 ± 5.18	10 (6, 15)
	Good (11–20)	283 (49.04)		
Total score for knowledge about HPV	Average (≤6)	274 (47.48)	7.98 ± 3.26	7 (5, 11)
	Good (7–12)	303 (52.51)		
Total score for knowledge about HPV vaccination	Average (≤2)	465 (80.58)	1.61 ± 0.95	1 (1, 2)
	Good (3–4)	112 (19.41)		
Total score for knowledge about HPV diagnosis	Average (≤1)	334 (57.88)	1.15 ± 1.16	1 (0, 2)
	Good (2–3)	243 (42.11)		
Age (years)		–	20.95 ± 1.82	21 (19, 22)

Abbreviations: BDS = Bachelor of Dental Surgery; HPV = Human papilloma virus; IQR = Interquartile range; MBBS = Bachelor of Medicine, Bachelor of Surgery; SD = Standard deviation.

Table 2 presents the descriptive statistics for knowledge about HPV, its vaccination, and diagnosis. Most students were aware of HPV (96.36%) and the associated diseases (94.28%). However, only 8.4% of the participants were aware of all the diseases caused by HPV, including cervical cancer, oropharyngeal cancer, vulvar cancer, and warts. While 34.14 % of the students correctly identified HPV 16 and 18 strains to be the cause for the majority of HPV-related cancers, only 28.24% were aware of all modes of its transmission (physical/sexual contact). Although 52.85% of the students knew that HPV could infect both genders, only 2% were aware of asymptomatic HPV infections. Moreover, 50.25% of the students had knowledge regarding the HPV vaccine, and 99.13% encouraged using such a vaccine among their close relatives. However, only 7.79% of these students realized the need for this vaccine in both genders in all the three age groups (9–13 years, 15–18 years, 20–26 years). Awareness regarding pap smear was also low (42.28%).

**Table 2:** Descriptive statistics for knowledge about HPV, its vaccination and diagnosis.

Variable			Frequency (%) [n = 577]

Are you aware of a virus called HPV?	Yes*		556 (96.36)
	No		21 (3.63)
Are you aware of any disease caused by HPV?	Yes*		544 (94.28)
	No		33 (5.71)
If your answer is yes to the above question, what disease/s does it cause?	Cervical cancer (A)		119 (23.28)
	Oropharyngeal cancer (B)		48 (9.39)
	Vulvar cancer (C)		29 (5.67)
	Warts (D)		97 (18.98)
	HIV/AIDS (E)		6 (1.17)
	A, B		65 (12.72)
	A, B, C		34 (6.65)
	A, B, C, D*		43 (8.41)
	B, D		12 (2.34)
	A, C, D		11 (2.15)
	B, C, D		16 (3.13)
	A, D		10 (1.95)
	A, C		11 (2.15)
	C, D		8 (1.56)
	A, B, D		22 (4)
Which HPV strains cause majority of HPV-related cancers?	HPV 16 & 18 (A)*		197 (34.14)
	HPV 6 & 11 (B)		92 (15.94)
	HPV 33 & 45 (C)		56 (9.7)
	None of the above		83 (14.38)
	A, B		91 (15.77)
	A, C		37 (6.41)
	B, C		21 (3.63)
Which of the following persons can be infected by HPV?	Male		65 (11.26)
	Female		196 (33.96)
	Both*		305 (52.85)
	Do not know		11 (1.9)
What is the mode of transmission of HPV?	Physical contact (A)		78 (13.51)
	Aerosol/Air droplet (B)		12 (2)
	Sexual intercourse (C)		266 (46.1)
	Do not know		32 (5.54)
	A, B		18 (3.11)
	A, C*		163 (28.24)
	B, C		8 (0.5)
Everyone infected with HPV will have symptoms.	True		205 (35.52)
	False*		12 (2)
	Do not know		266 (46.1)
Cervical cancer is a leading cause of cancer deaths among women of India.	True		255 (44.19)
	False*		295 (51.12)
	Do not know		27 (4.67)
Are you aware that HPV can also cause oropharyngeal carcinoma?	Yes*		308 (53.37)
	No		269 (46.62)
Are you aware of any vaccine to prevent these virus-related diseases?	Yes*		290 (50.25)
	No		287 (49.74)
Is there a need for better education in India about HPV and its related diseases?	Yes*		570 (98.78)
	No		7 (1.21)
Do you feel there will be an increased trend of HPV-related diseases in the near future?	Yes*		571 (99.13)
	No		6 (1.03)
Do you wish to gain more knowledge about HPV and its related diseases?	Yes*		573 (99.3)
	No		4 (0.69)
Do you know the name of the vaccine given to prevent HPV related diseases?	Yes*		200 (34.66)
	No		377 (65.33)
What age group should receive this vaccine?	9–13 years (A)	Girls	12 (2.07)
		Boys	0 (0)
		Both*	6 (1.03)
	15–18 years (B)	Girls	20 (3.46)
		Boys	0 (0)
		Both*	68 (11.78)
	20–26 years (C)	Girls	116 (20.1)
		Boys	0 (0)
		Both*	113 (19.58)
	Do not know		38 (6.58)
	A, B (Girls)		0 (0)
	B, C (Girls)		42 (7.27)
	A, C (Girls)		0 (0)
	A, B (Both)		15 (2.59)
	A, C (Both)		19 (3.29)
	B, C (Both)		52 (9.01)
	A, B, C (Girls)		31 (5.37)
	A, B, C (Both)*		45 (7.79)
Are you aware that HPV vaccines, unlike in other countries, are still not a part of the National Immunization Program of India?	Yes*		125 (21.66)
	No		452 (78.33)
Would you allow your child or a close relative to get HPV vaccination?	Yes*		572 (99.13)
	No		0 (0)
	Maybe		5 (0.86)
Have you heard about Pap smear test?	Yes*		333 (42.28)
	No		244 (57.71)
What is a Pap smear test used for?	Testing sexually transmitted diseases		255 (44.19)
	Treating cervical cancer		151 (26.16)
	Cervical cancer screening*		117 (20.27)
	Do not know		54 (9.35)
Is there a need for Pap smear screening after receiving HPV vaccination?	True*		237 (41.07)
	False		126 (21.83)
	Do not know		214 (37.08)

Abbreviations: * Correct answer; HPV = Human papilloma virus.

Table 3 depicts between-course and between-gender analyses. Using the Mann Whitney U test, no significant difference was observed between dental and medical students as well as between males and females in the distribution of overall score as well as the total scores for knowledge about HPV, its vaccination, and its diagnosis (p > 0.05).

**Table 3:** Between-course and between-gender analysis.

Variable	Between-Course Analysis					Between-Gender Analysis				

	Dental (BDS)		Medical (MBBS)		p-value	Female		Male		p-value
	
	Median (Range)	Mean ± SD	Median (Range)	Mean ± SD		Median (Range)	Mean ± SD	Median (Range)	Mean ± SD	

Overall score	10.5 (6, 15)	10.9 ± 5.23	10 (6, 14)	10.59 ± 5.12	0.41	11 (6, 15)	10.97 ± 5.19	8 (6, 14)	10.44 ± 5.15	0.24
Total score for knowledge about HPV	8 (5, 11)	8.2 ± 3.4	7 (5, 10)	7.73 ± 3.09	0.06	9 (5, 11)	8.18 ± 3.27	6 (5, 11)	7.7 ± 3.24	0.14
Total score for knowledge about HPV vaccination	1 (1, 2)	1.56 ± 0.89	1 (1, 2)	1.67 ± 1.02	0.28	1 (1, 2)	1.63 ± 1.01	1 (1, 2)	1.57 ± 0.86	0.89
Total score for knowledge about HPV diagnosis	1 (0, 2)	1.13 ± 1.14	1 (0, 2)	1.18 ± z1.19)	0.68	1 (0, 2)	1.15 ± 1.13	1 (0, 2)	1.17 ± 1.21	0.95

Abbreviations: BDS = Bachelor of Dental Surgery; HPV = Human papilloma virus; MBBS = Bachelor of Medicine, Bachelor of Surgery; SD = Standard deviation.

Tables 4 and 5 present the comparison of variables between the courses and the genders, respectively. Using Chi-square test, neither the male/female genders nor the dental/medical courses showed any significant association with the overall score as well as the total scores for knowledge about HPV, its vaccination, and its diagnosis (p > 0.05). However, the total score for knowledge about HPV was significantly greater in females compared to males (p = 0.006).

**Table 4:** Comparison of variables between the courses.

Variable		Course		p-value

		BDS	MBBS	

Overall score	Average (≤10)	152 (50)	142 (52.01)	0.68
	Good (11–20)	152 (50)	131 (47.98)	
Total score for knowledge about HPV	Average (≤6)	142 (46.71)	132 (48.35)	0.75
	Good (7–12)	162 (53.28)	141 (51.64)	
Total score for knowledge about HPV vaccination	Average (≤2)	243 (79.93)	222 (81.31)	0.75
	Good (3–4)	61 (20.06)	51 (18.68)	
Total score for knowledge about HPV diagnosis	Average (≤1)	182 (59.86)	152 (55.67)	0.35
	Good (2–3)	122 (40.13)	121 (44.32)	

Abbreviations: BDS = Bachelor of Dental Surgery; HPV = Human papilloma virus; MBBS = Bachelor of Medicine, Bachelor of Surgery.

**Table 5:** Comparison of variables between the genders.

Variable		Gender		p-value

		Female	Male	

Overall score	Average (≤10)	163 (48.51)	131 (54.35)	0.19
	Good (11–20)	173 (51.48)	110 (45.64)	
Total score for knowledge about HPV	Average (≤6)	143 (42.55)	131 (54.35)	0.006*
	Good (7–12)	193 (57.44)	110 (45.64)	
Total score for knowledge about HPV vaccination	Average (≤2)	264 (78.57)	201 (83.40)	0.18
	Good (3–4)	72 (21.42)	40 (16.59)	
Total score for knowledge about HPV diagnosis	Average (≤1)	194 (57.73)	140 (58.09)	0.99
	Good (2–3)	142 (42.26)	101 (41.9)	

Abbreviations: * Significant at 5% level of significance; HPV = Human papilloma virus.

## Discussion

India is the primary contributor to the global burden of HPV infection and associated diseases like CC and oropharyngeal cancer. Hence, it is essential to recognize the existing knowledge pool of current healthcare students about HPV and its preventive measures to translate this into benefits for the society in the future. Therefore, the present study was conducted to determine the awareness and knowledge among dental and medical undergraduate students regarding HPV and its diagnosis and prevention.

Like the current research, Radhika et al. evaluated the awareness and knowledge of the HPV vaccine in the prevention of CC among medical students [7]. They found that most of the participants were aware about the causal association between HPV and CC (80%), the preventable nature of CC (76%) as well as the availability of HPV vaccines (72%) [7]. However, they expressed lower awareness regarding the cost (30%) and the efficacy (10%) of these vaccines [7]. Over 96% students had a positive attitude towards educating people regarding HPV vaccination, closely mirroring the present study (99.13%) [7].

A similar study was conducted by Mehta et al., who observed a lack of knowledge among medical students regarding HPV infection and its association with sexually transmitted diseases like CC (50%), the correct incidence of CC in India (100%), and prevention of CC by HPV vaccination (18%). They also possessed a negative attitude towards preventive measures, with 50% of the students assuming that vaccination merely induced a false sense of security [11]. Compared to this research, the present study revealed poorer awareness but a more positive attitude among the healthcare students regarding HPV and its prevention.

Challa et al., too, conducted a similar study and reported that 100% of the medical students were aware of the causal association between CC and HPV, 95% knew that CC could be prevented, 81.1% were aware of the sexual route of HPV transmission, 78.7% knew that pap smear could detect HPV infection and 77.9% knew that HPV vaccine is available in India, in contrast to the present study (23.28%, 50.25%, 46.1%, 20.27%, and 34.66%, respectively), reflecting poorer knowledge [12].

Analogous research by Pandey et al. revealed that 89.2% of the medical students identified HPV as the etiology behind CC, 89.6% acknowledged the preventive nature of CC, 75.6% knew of CC vaccine availability, 86.2% expressed interest in expert education in this regard and 67.8% showed acceptance towards the HPV vaccine [13].

To the best of the authors’ knowledge, no study could be found that compared HPV or CC awareness between dental and medical students. However, Challa et al. noted that females showed better awareness and acceptance of the CC vaccine (p < 0.05), similar to the present study, whereby knowledge about HPV was significantly greater in females compared to males (p = 0.006), though this difference was not reflected in awareness regarding vaccination and diagnosis of HPV [12].

A cross-sectional study by Sallam et al. found gaps in knowledge regarding HPV-related oral cancer among dental students, thus necessitating interventions such as curricular changes, training workshops, and awareness campaigns [14]. Nearly 88% of the students correctly identified HPV as a risk factor for oropharyngeal cancer development, compared to only 9.39% in the present study [14].

Another study by Kepka et al. revealed that up to 83.7% of oral health students believed that administering HPV knowledge and vaccination fell within the scope of practice of dental professionals, encouraging their participation in eradicating this virus [15].

The results of the present research uncover the need for more integrated teaching regarding HPV carcinogenesis, CC, its diagnosis, and vaccination among healthcare students. This would promote the awareness and acceptance among the masses with respect to HPV screening and immunization, dispelling myths and misconceptions. This would help in the improvement of the quality of life of the population at large.

However, this research has its limitations in being a single-center study with limited sample size. The information regarding the mechanism of action, dosage, schedule, and cost of the HPV vaccine was also not evaluated. Moreover, the translation of student knowledge to clinical and social practice over time is difficult. These limitations can be overcome by multicentric, long-term, prospective clinical studies with a larger sample size evaluating a wider array of parameters.

## Conclusion

Overall, the dental and medical undergraduate students presented an average level of knowledge and awareness regarding HPV, as well as its diagnosis and prevention. This reflects a greater need for educating HCP in order to have a ripple effect on society at large.

## References

[1] World Health Organization. Human papillomavirus (HPV) and cervical cancer. https://www.who.int/news-room/fact-sheets/detail/human-papillomavirus-(hpv)-and-cervical-cancer.

[2] Elrefaey S, Massaro MA. HPV in oropharyngeal cancer: The basics to know in clinical practice. Acta Otorhinolaryngol Ital. 2014; 34(5): 299–309. 25709145 PMC4299160

[3] Lowy DR, Schiller JT. Reducing HPV-associated cancer globally. Cancer Prev Res (Phila). 2012; 5(1): 18–23. DOI: 10.1158/1940-6207.CAPR-11-0542 22219162 PMC3285475

[4] Parkin DM, Bray F. Estimating the world cancer burden: Globocan 2000. Int J Cancer. 2001; 94: 153–6. DOI: 10.1002/ijc.1440 11668491

[5] Mehta S, Rajaram S. Awareness about Human Papilloma Virus and its vaccine among medical students. Indian J Community Med. 2013; 38(2): 92–4. DOI: 10.4103/0970-0218.112438 23878421 PMC3714948

[6] Canadian Immunization Committee. Summary of Canadian Immunization Committee (CIC) recommendations for human papilloma virus immunization programs. Can Commun Dis Rep. 2014; 40(8): 152–3. DOI: 10.14745/ccdr.v40i08a02 29769896 PMC5864433

[7] Radhika M, Sadiqunissa. Awareness and knowledge of human papilloma virus (HPV) vaccine in prevention of cervical cancer among medical students. Int J Reprod Contracept Obstet Gynecol. 2018; 7: 5026–30. DOI: 10.18203/2320-1770.ijrcog20184960

[8] Markowitz LE, Dunne EF, Saraiya M, et al. Quadrivalent Human Papilloma virus Recommendation of the Advisory Committee on Immunization Practices (ACIP) MMWR Recomm Rep. 2007; 56: 1–24. DOI: 10.1037/e601292007-001 17380109

[9] Sankaranarayanan R, Bhatla N. Current global status & impact of human papillomavirus vaccination: Implications for India. Indian J Med Res. 2016; 144(2): 169–80. DOI: 10.4103/0971-5916.195023 27934795 PMC5206867

[10] Goldie SJ, O’Shea M. Benefits, cost requirements and cost-effectiveness of the HPV 16, 18 vaccine for cervical cancer prevention in developing countries: Policy implications. Reprod Health Matters. 2008; 16: 86–96. DOI: 10.1016/S0968-8080(08)32409-4 19027626

[B11] Mehta S, Rajaram S. Awareness about human papilloma virus and its vaccine among medical students. Indian J Community Med. 2013; 38(2): 92–4. DOI: 10.4103/0970-0218.112438 23878421 PMC3714948

[B12] Challa N, Madras V. Awareness and attitude regarding human papilloma virus and its vaccine among medical students in a medical school in India. Int J Res Med Sci. 2014; 2: 1607–11. DOI: 10.5455/2320-6012.ijrms20141168

[B13] Pandey D, Vanya V. Awareness and attitude towards human papillomavirus (HPV) vaccine among medical students in a premier medical school in India. PLoS One. 2012; 7(7): e40619 DOI: 10.1371/journal.pone.0040619 22859950 PMC3409219

[14] Sallam M, Al-Fraihat E. Dental students’ awareness and attitudes toward HPV-related oral cancer: A cross sectional study at the University of Jordan. BMC Oral Health. 2019; 19(1): 171 DOI: 10.1186/s12903-019-0864-8 31370845 PMC6670240

[15] Kepka D, Rutkoski H, Pappas L, et al. US oral health students’ willingness to train and administer the HPV vaccine in dental practices. Prev Med Rep. 2019; 15: 100957 DOI: 10.1016/j.pmedr.2019.100957 31372330 PMC6661379

